# Alpha power increases in right parietal cortex reflects focused internal attention

**DOI:** 10.1016/j.neuropsychologia.2014.02.010

**Published:** 2014-04

**Authors:** Mathias Benedek, Rainer J. Schickel, Emanuel Jauk, Andreas Fink, Aljoscha C. Neubauer

**Affiliations:** Department of Psychology, University of Graz, Steiermark 8010, Austria

**Keywords:** Attention, Memory, Inhibition, EEG, Alpha, Divergent thinking

## Abstract

This study investigated the functional significance of EEG alpha power increases, a finding that is consistently observed in various memory tasks and specifically during divergent thinking. It was previously shown that alpha power is increased when tasks are performed in mind—e.g., when bottom-up processing is prevented. This study aimed to examine the effect of task-immanent differences in bottom-up processing demands by comparing two divergent thinking tasks, one intrinsically relying on bottom-up processing (sensory-intake task) and one that is not (sensory-independence task). In both tasks, stimuli were masked in half of the trials to establish conditions of higher and lower internal processing demands. In line with the hypotheses, internal processing affected performance and led to increases in alpha power only in the sensory-intake task, whereas the sensory-independence task showed high levels of task-related alpha power in both conditions. Interestingly, conditions involving focused internal attention showed a clear lateralization with higher alpha power in parietal regions of the right hemisphere. Considering evidence from fMRI studies, right-parietal alpha power increases may correspond to a deactivation of the right temporoparietal junction, reflecting an inhibition of the ventral attention network. Inhibition of this region is thought to prevent reorienting to irrelevant stimulation during goal-driven, top-down behavior, which may serve the executive function of task shielding during demanding cognitive tasks such as idea generation and mental imagery.

## Highlights

• This study investigated the functional significance of EEG alpha activity.
• Right-parietal alpha power increased as a function of internal attention demands.
• Alpha power increases during divergent thinking indicates focused attention.
• Right-parietal alpha may reflect activity of the ventral attention network.

## 1 Introduction

EEG alpha activity is the dominant oscillatory activity of the human brain (Niedermeyer & Lopes da Silva, 1999). It has been associated with basic cognitive functions such as attention or memory (Klimesch, 2012), and also with more complex cognitive processes such as divergent thinking (i.e., creative idea generation; Fink and Benedek, 2013, in press). A recent experimental study found that a prevention of bottom-up information processing causes alpha power increases in convergent and divergent thinking tasks (Benedek, Bergner, Könen, Fink, & Neubauer, 2011). The present study aims to follow up these findings to disentangle alpha effects as a cause of experimentally enforced internal attention, and due to task-dependent attention demands.

EEG research has a long tradition in studying oscillatory brain activity related to various cognitive tasks and emotional states. This led to the identification of different frequency bands within the EEG power spectrum, such as alpha, beta, gamma or theta, which proved to be sensitive to discriminable psychological functions (e.g., Klimesch, 1999; Fries, 2005; von Stein & Sarntheim, 2002). The investigation of alpha activity (8–12 Hz) led to some controversy about its functional significance. The frequent observation that alpha activity shows task-related decreases in various cognitive tasks (i.e., alpha desynchronization) but increases (i.e., alpha synchronization) during rest and with eyes closed, led to the notion that alpha activity reflects a cognitive default state such as ‘cortical idling’ (Pfurtscheller, Stancak, & Neuper, 1996). Other studies observing task-related increases of alpha activity e.g., during memory retention (Klimesch, Doppelmayr, Schwaiger, Auinger, & Winkler, 1999), or with increasing task load (Jensen, Gelfand, Kounios, & Lisman, 2002), however, suggest a more active role of alpha activity.

Examining the functional significance of EEG alpha and beta activity, Ray and Cole (1985) found that alpha power is lower in *sensory-intake* tasks (i.e., tasks that rely on processing of external stimuli, such as counting verbs in a passage or the paper folding task) as compared to *intake-rejection* tasks (i.e., tasks that do not require processing of external sensory stimuli, such as mental arithmetic or imagination of an imaginary walk). They suggested that alpha activity reflects attentional demands and is higher for tasks with internal attention focus than for tasks with external attention focus. Other research using short-term memory tasks found alpha activity to increase as a function of memory load (Jensen et al., 2002; Klimesch et al., 1999). It was proposed that alpha increases may reflect active top-down inhibition of task irrelevant brain regions, such as inhibition of access to semantic long-term memory (Klimesch et al., 1999), or inhibition or disengagement of visual areas to suppress the processing of irrelevant visual information (Jensen et al., 2002). The latter interpretation is supported by findings showing alpha increases over occipital cortex contralateral to the position of distractor stimuli in spatial cuing paradigms (Händel, Haarmeier, & Jensen, 2011; Rihs, Michel, & Thut, 2007; Worden, Foxe, Wang, & Simpson, 2000).

Another function that has been attributed to alpha activity is that phase coherence in the alpha range between different brain regions may be an important mechanism underlying intracortical interaction such as top-down control (Engel, Fries, & Singer, 2001; Von Stein & Sarnthein, 2000; Zanto, Rubens, Thangavel, & Gazzaley, 2011). Moreover, it was suggested that the phase characteristics of alpha activity reflect a mechanism of functional inhibition at neuronal level that supports rhythmic updating (Chakravarthi & VanRullen, 2012), gating of information (Jensen & Mazaheri, 2010), and phase coding of information (Jensen, Bonnefond, & VanRullen, 2012). Klimesch (2012) proposed that alpha activity has both roles: inhibition of task-irrelevant networks and timing within task-relevant networks. Alpha activity thus plays an important role for attention by supporting processes within the attentional focus and blocking processes outside its focus.

Over the last years, task-related increases in alpha activity have also been consistently observed during performance of divergent thinking tasks (i.e., creative idea generation tasks; Fink and Benedek, 2013, in press). For example, in the alternate uses task (a task also commonly used in psychometric research on creative potential; Benedek, Mühlmann, Jauk, and Neubauer, 2013; Kaufman, Plucker, and Baer, 2008) participants are asked to generate creative new uses for common objects such as a “shoe”. Performance of this and other divergent thinking tasks consistently results in task-related power (TRP) increases in the alpha band as compared to a pre-task reference period. Alpha synchronization was found to be strongest in frontal brain regions but also high in posterior parts of the right hemisphere (Fink & Benedek, in press). A number of EEG studies further revealed that EEG alpha activity is sensitive to creativity-related demands of tasks (more alpha in task showing higher as compared to lower free-associative, divergent thinking; e.g., Jauk, Benedek, and Neubauer (2012), Jaušovec (1997) to creativity of ideas (more alpha for more as compared to less creative ideas; Fink and Neubauer, 2006; Grabner, Fink, and Neubauer, 2007), to individual differences in creativity (more alpha in more creative people; Fink and Neubauer, 2008; Fink et al., 2009a,b); Jaušovec, 2000; Martindale and Hines, 1975; Martindale and Hasenfus, 1978), and to increase after successful creativity-enhancing interventions (Fink, Grabner, Benedek, & Neubauer, 2006; Fink, Schwab, & Papousek, 2011). These findings suggest that creative cognition is reliably associated with increased alpha power levels in the brain (for a review, see Fink and Benedek, 2013, in press).

Considering the evidence on the functional significance of alpha activity, it yields the question to what extent alpha activity during divergent thinking is either due to processes specific for creative cognition, or due to more general (e.g., attentional) demands of these tasks. This question has recently been addressed in an EEG study varying creative cognition-related task demands (convergent vs. divergent thinking) and attentional task demands (low vs. high internal attention demands) as experimental factors in a within-subject design (Benedek et al., 2011). In the convergent thinking task participants had to solve four-letter anagram problems which have just one correct solution; in the divergent thinking task participants were presented the same four-letter words but had to generate original four-word sentences with the letters as initials. Additionally, stimuli either remained visible throughout the task, or were masked after 500 ms to avoid any further bottom-up information processing. The latter condition was intended to implement higher internal attention demands. A comparison of task-related alpha power between tasks and conditions showed that alpha power increases were particularly related to high internal attention demands, rather than differences between tasks. During high internal attention demands alpha synchronization was observed in both tasks especially at frontal sites, and for the divergent thinking task also at posterior parietal sites of the right hemisphere. During low internal attention demands, however, both tasks showed task-related decreases of alpha power. This finding supports the notion of alpha activity reflecting internal attention.

What is still unclear, however, is the question why in this study in the divergent thinking task alpha synchronization was only observed when high internal attention demands were experimentally induced, although it had been observed in many previous studies for divergent thinking without any stimulus masking (Fink & Benedek, in press). It was proposed that this may be due to the nature of the employed divergent thinking task that was specifically adapted for this study (Benedek et al., 2011): Generating four-word sentences from four letters may rely on the processing of external information as four abstract stimuli have to be considered and manipulated. Most other divergent thinking tasks, however, encode and process verbal stimuli as single concepts and thus may not require further bottom-up processing during the task. We assume that the amount of task-related alpha activity during divergent thinking does not only depend on the availability of relevant external information but particularly on whether the task requires that attention is continuously directed to the processing of external information or not.

To test this hypothesis, we performed another experiment similar to the previous one, but this time contrasting two types of divergent thinking (DT) tasks—one DT task involving the processing of external information, whereas the other one is not. These tasks could be categorized as *sensory-intake* and *sensory-independence* (or intake-rejection; Ray and Cole, 1985) tasks. For the sensory-intake task, we again employed the four-word sentence generation task. This task was shown to involve processing of external information since performance decreases after stimulus masking (Benedek et al., 2011). For the sensory-independence task, we employed the alternate uses task, a widely used divergent thinking task which requires generating creative uses of common objects. In both tasks we presented four-letter words denoting objects. In the four-word sentence task this stimulus is processed as four abstract elements of information, whereas in the alternate uses task it is processed as one conceptual stimulus. Additionally, as in the previous study, both tasks were performed with the stimulus either remaining visible (low internal attention condition) or being masked directly after encoding (high internal attention condition). We hypothesized that the stimulus masking would predominantly affect the sensory-intake task which typically relies on processing of external information, leading to higher alpha power in the high as compared to the low internal attention condition. In contrast, stimulus masking should not affect the sensory-independent task as it does not rely on processing of external information. Finally, since the sensory-independence task naturally shows focused internal attention, it should show higher alpha power than the sensory-intake task especially in the low internal attention condition.

## 2 Methods

### 2.1 Participants

40 students (20 female) participated in this study. On average, participants were 25.4 years old (SD=2.87; range=20–32 years). All participants were right-handed, had normal or corrected-to-normal vision and reported no medical or psychological disorders. Participants gave written informed consent prior to the EEG recording session. The procedure was approved by the Ethics Committee of the University of Graz.

### 2.2 Experimental tasks and conditions

Two divergent thinking tasks were employed in this study, the four-word sentences (FS) task and the alternate uses (AU) task. In both tasks, single four-letter words denoting an object (e.g., “SHOE”) were presented as stimuli. In the FS task, participants were required to create an original but meaningful four-word sentence using the given four letters of the stimulus word as initial letters (e.g., for the item “SHOE” a possible response would be “Superman hates evil operations”). As shown in this example, the order of the initial letters in the sentence can differ from the order of letters in the stimulus word, but every letter has to be used exactly once. This task was adapted from a well-known German creativity test (Verbaler Kreativitäts-Test; VKT, Schoppe, 1975), and it was also used in a previous EEG study (Benedek et al., 2011). In the AU task, participants were required to find a creative use for the presented objects (e.g., for the item “SHOE” a possible response would be “use it as a flower pot”). The AU task is one of the most commonly used divergent thinking tasks in psychometric and neuroscientific research (Fink & Benedek, 2013). Both tasks are considered divergent thinking tasks as there are nearly unlimited possible valid solutions for every stimulus (Guilford, 1967). However, the tasks differed in the way the stimuli had to processed, either focusing on the four letters of the stimulus word (in the FS task), or on the concept represented by the stimulus word (in the AU task). Though the FS task can generally be mastered without continuous access to the stimulus, this involves a considerable taxing of working memory, as indicated by performance decreases compared to a visible condition (Benedek et al., 2011). When the stimulus remains visible, not all stimulus elements have to be kept in working memory throughout the task as they can be continuously accessed on demand (Benedek et al., 2011). The FS task thus can be conceived as a *sensory-intake* task as it intrinsically involves the processing of external information. In contrast, in the AU task the stimulus is encoded as a single conceptual stimulus. A single conceptual stimulus can be easily kept in mind, and it can be assumed that a continuous processing of this stimulus would not help much to relieve working memory but would rather interfere with ongoing processes. Since this task does not intrinsically involve the processing of external information it can be considered as a *sensory-independence* task.

As a second experimental factor, both tasks were presented in two different presentation conditions involving either low or high internal processing (i.e., internal attention) demands. In the low internal processing (LIP) condition, the stimuli were kept visible on screen thus allowing the participants to process the stimulus characters during the entire task in a bottom-up manner. In contrast, in the high internal processing (HIP) condition, stimuli were presented for 500 ms and then masked by “XXXX”. This supraliminal stimulus presentation is sufficiently long to allow for encoding of the meaningful stimulus word, but too short for solving the task. The latter experimental condition is assumed to require higher internal processing demands, as the problem has to be solved without further bottom-up processing of the stimulus. Both experimental factors were varied within-subject resulting in a 2×2 within-subject design.

A stimulus set of 20 four-letter German words denoting familiar objects was compiled for this study (VASE, DOSE, KORB, BETT, BUCH,BALL, TOPF, RING,HELM, ZELT, TUCH, BEIL, MEHL, HOSE, BROT, STAB, SARG, LUPE, SEIL, SIEB [vase, can, basket, bed, book, ball, pot, ring, helmet, tent, rag, axe, flour, trousers, bread, stick, coffin, magnifier, rope, colander]). These stimuli were used in both tasks (FS and AU), thus avoiding any systematic differences in physical stimulus complexity between tasks. For each task, all 20 stimuli were used once and presented in an individually randomized sequence, with half of the items (10 items) randomly assigned to the LIP condition and the other half to the HIP condition. This ensured that participants could not anticipate content or masking condition for any stimulus.

**Fig. 1:**
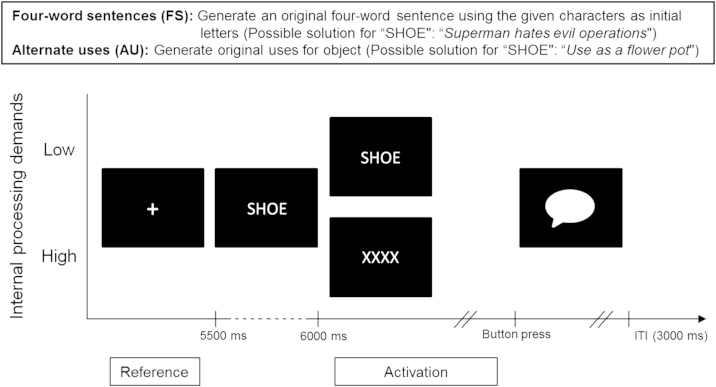
Above: Brief task description of the four-word sentences task and the alternate uses task. Below: Trial sequence for the two experimental conditions. The fixation cross was followed by the presentation of the stimulus. In half of trials the stimulus remained visible throughout the task (low internal processing demands), and in the other half the stimulus was masked after 500 ms (high internal processing demands). When came to a response they pressed a button, and then were asked to express their response vocally. Trials were separated by an inter-trial-interval (ITI) of 3000 ms.

The trial sequence was the same for both tasks types. Each trial started with the presentation of a white fixation cross for 5 s. This was followed by a stimulus word presented in white capital letters in the center of the screen. Participants were asked to generate one single response. Moreover, they were required to keep eyes open during idea generation. As soon as the participants came up with a response they pressed a button and then were prompted to vocalize the response. Responses were recorded with a microphone, and transliterated after the EEG session. After the response participants had to push the button again in order to proceed to the next trial. The timeout duration per trial was set to 45 s. All trials were interspersed with 3 s inter-trial-intervals (ITI; see Fig. 1).

### 2.3 Data acquisition and analysis

The EEG was recorded by means of a customary EEG amplifier (BrainAmp and Vision Recorder 1.20; Brain Products, Gilching, Germany) and sampled at a frequency of 500 Hz. Gold electrodes (9 mm diameter) were located in an electrode cap in 33 positions (according to the international 10–20 system with interspaced positions), a ground electrode was located on the forehead, the reference electrode was placed on the nose. To register eye movements, an electrooculogram (EOG) was recorded bipolarly between two gold electrodes diagonally placed above and below the inner respectively the outer canthus of the right eye. The EEG signals were filtered between 0.1 Hz and 100 Hz; an additional 50 Hz notch filter was applied to avoid power line contamination. Electrode impedances were kept below 5 kΩ for the EEG and below 10 kΩ for the EOG. The EEG signal was corrected for ocular artifacts by means of an automated regression-based method (Gratton, Coles, & Donchin 1983; Vision Analyzer 1.05, Brain Products, Gilching, Germany), and by means of a subsequent visual inspection of possible remaining artifacts caused by eye blinks, eye movements or muscle tension, which were marked and excluded from further analyses. In a next step, the band power of the EEG signal was computed by means of a time–frequency analysis employing a standard FFT applied to time windows of 1000 ms with 900 ms overlap. From this, the power in the alpha frequency band (8.5–12.5 Hz) was extracted. Since previous studies repeatedly revealed consistent results for the lower and upper alpha band (e.g., Benedek et al., 2011; Jauk et al., 2012; see also Fink and Benedek, in press) we did not perform additional analyses for alpha sub-bands in this study. For complementary analyses we additionally computed band power for the theta frequency band (4.5–6.5 Hz) and the beta frequency band (15.5–30.5 Hz).

Brain activity during the performance of experimental tasks was quantified by means of task-related power (TRP) changes in the EEG (Pfurtscheller, 1999). Task-related power at an electrode *i* was obtained by subtracting the log-transformed power during prestimulus reference intervals from the log-transformed power during the activation intervals according to the formula: TRP(i)=log(Pow_*i*_, activation)—log(Pow_*i*_, reference). Decreases in power from the reference to the activation interval hence are expressed as negative values (i.e., alpha desynchronization), whereas task-related increases in power (i.e., alpha synchronization) are expressed as positive values. As shown in Fig. 1, a 4-s time interval during presentation of the fixation cross (500 ms to 4500 ms after onset of the fixation cross) served as pre-stimulus reference interval for TRP calculation. In both tasks types (sensory-intake and sensory-independence task) and both experimental conditions (LIP and HIP) the activation interval was defined to range from 1000 ms after stimulus onset to 500 ms before the pressing of the idea button, restricted to a maximum activation period of 30 s (see Fig. 1; it should be noted that the average response time was 27 s and did not differ between tasks, see Section 3). By defining the activation period to start not until 1000 ms after stimulus onset (or 500 ms after stimulus masking in the HIP condition), the TRP is reflects task performance but not initial stimulus encoding or masking. Only trials with valid responses, and consisting of artifact-free data of more than 500 ms in the reference and the activation periods were included in further analyses. Moreover, participants who fail to show a minimum of three valid trials in all tasks and conditions would be excluded from further analyses. All participants met these criteria and thus were retained in the analyses.

For statistical analyses, electrode positions were topographically aggregated as following: anteriofrontal (AF) left (FP1, AF3), frontal (F) left (F3, F7), frontocentral (FC) left (FC1, FC5), centrotemporal (CT) left (C3, T3), centroparietal (CP) left (CP1, CP5), parietotemporal (PT) left (P3, T5), parietooccipital (PO) left (PO3, PO5, O1), and analogously for the right hemisphere. The midline electrodes (FZ, CZ, PZ) were not included in the analyses as we were also interested in hemispheric differences. All analysis settings matched those of a previous study (Benedek et al., 2011) to ensure comparability.

### 2.4 Procedure

The participants were seated comfortably in the darkened sound-attenuating EEG recording room, electrodes were mounted and impedances checked. In the beginning of the experiment two 2-min EEG sequences under resting conditions were recorded, the first one with eyes closed, the second one with eyes open. Then the two EEG tasks were presented in randomized sequence, either starting with the FS or the AU task. Prior to each task, participants were familiarized with task requirements and response mode by performing four exercise trials. The EEG session lasted about one hour in total.

## 3 Results

### 3.1 Behavioral results

**Fig. 2:**
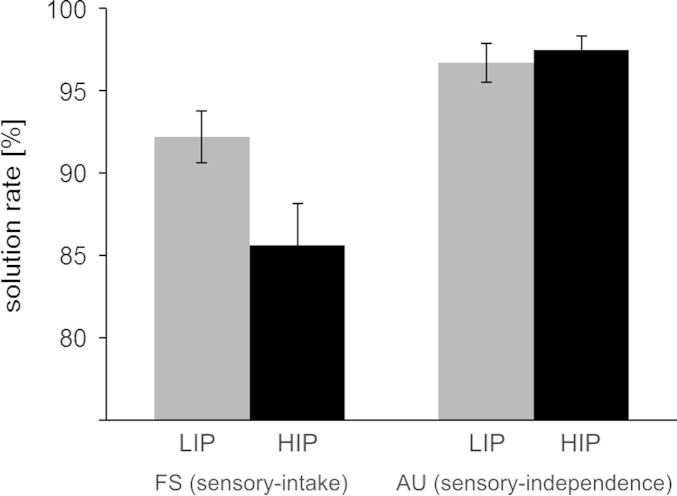
Solution rate in the four-word sentences (FS) task (i.e., sensory-intake task) and the alternate uses (AU) task (i.e., sensory-independence task). Both task were performed under two experimental conditions with either low internal processing (LIP) demands (=stimulus remained visible), or higher internal processing (HIP) demands (=stimulus was masked).

Task performance was analyzed with respect to the solution rate (i.e., relative number of correct responses) and response time (of correct trials) by means of ANOVAs considering the within-subject factors TASK (FS vs. AU) and experimental condition (COND: low vs. high internal processing demands; LIP vs. HIP). All tasks and conditions showed a high solution rate of >85%. A significant TASK effect (*F*(1,39)=28.34, *p*=.001, partial-*η*^2^=.42), a nearly significant condition effect (*F*(1,39)=3.86, *p*=.06, partial-*η*^2^=.09), and an interaction of TASK⁎COND (*F*(1,39)=5.78, *p*=.02, partial-*η*^2^=.13) indicated that the solution rate in the FS task (i.e., sensory-intake task) was lower than in the AU task i.e., sensory-independence task), particularly when the former was performed in the HIP condition (see Fig. 2). Tasks and conditions, however, did not differ in their response time which was on average 27 s (TASK: *F*(1,39)=0.02, *p*=.89; COND: *F*(1,39)=0.12, *p*=.73; TASK⁎COND: *F*(1,39)=0.04, *p*=.84).

### 3.2 EEG results

**Fig. 3:**
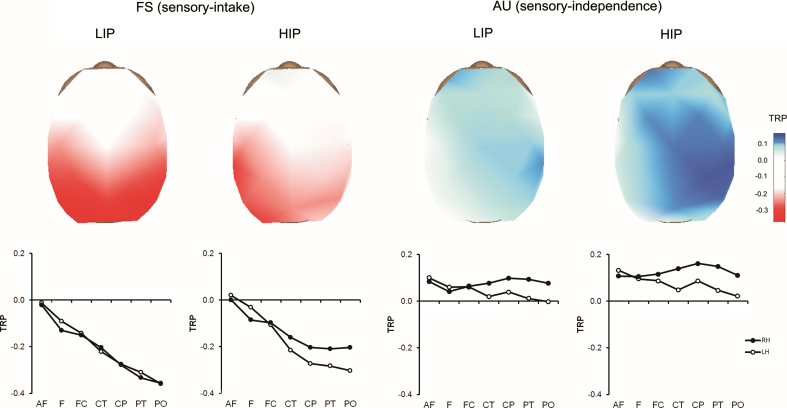
Brain maps showing task-related power (TRP) in the alpha band (8.5–12.5 Hz) in the four-word sentences (FS) task (i.e., sensory-intake task) and the alternate uses (AU) task (i.e., sensory-independence task). Both task were performed under two experimental conditions with either low internal processing (LIP) demands (=stimulus remained visible), or higher internal processing (HIP) demands (=stimulus was masked). Positive TRP indicates task-related alpha synchronization, negative values indicate desynchronization (AF=anteriofrontal, F=frontal, FC=frontocentral, CT=centrotemporal, CP=centroparietal, PT=parietotemporal, PO=parietooccipital; LH=left hemisphere, RH=right hemisphere).

Task-related power (TRP) changes in the alpha band were analyzed by means of ANOVAs for repeated measures considering the within-subject factors TASK (FS, sensory-intake vs. AU, sensory-independence), experimental condition (COND: LIP vs. HIP), hemisphere (HEMI: left vs. right) and AREA (anteriofrontal, frontal, frontocentral, centrotemporal, centroparietal, parietotemporal, and parietooccipital). A multivariate analysis approach (Pillai׳s trace) was employed which is known to be robust in case of violations of sphericity (Vasey & Thayer, 1987). Tukey׳s HSD posttests were computed to further examine significant effects. This 2×2×2×7 ANOVA revealed a significant TASK effect (*F*(1,39)=29.13, *p*=.001, partial-*η*^2^=.43) which interacted with AREA (*F*(6,34)=6.47, *p*=.001, partial-*η*^2^=.44) indicating that the AU task showed higher task-related alpha increases (i.e., positive TRP, or alpha synchronization) in posterior regions of the brain than the FS task, which showed decreases of task-related alpha power (i.e., negative TRP, or alpha desynchronization). Moreover, we observed a weak tendency towards an TASK⁎COND⁎AREA effect (*F*(6,34)=1.91, *p*=.11, partial-*η*^2^=.25) suggesting that condition effects in posterior brain regions were specific to the FS rather than the AU task (see Fig. 1). Since high-factorial designs, as this 2×2×2×7 ANOVA, usually have low power for testing higher-order interactions, we further explored effects separately for each task. For the FS task, we observed a significant condition effect in terms of a significant interaction of COND⁎AREA⁎HEMI (*F*(6,34)=2.81, *p*=.03, partial-*η*^2^=.33). As illustrated in Fig. 3, the FS task resulted in higher task-related alpha power (i.e., lower alpha desynchronization) in parietal and occipital regions of the right hemisphere (*p*s<.002 in CP, PT, PO regions) in the HIP condition, but not in the LIP condition (all *p*s>.6). Further significant effects of AREA (*F*(6,34)=7.21, *p*=.001, partial-*η*^2^=.56) and AREA⁎HEMI (*F*(6,34)=2.95, *p*=.02, partial-*η*^2^=.34) point to general topographic characteristics of the TRP activation pattern in the FS task, which are part of the three-way interaction including the factor COND.

Considering the AU task, no significant condition effects were observed, besides a weak tendency towards a main effect COND (*F*(1,39)=2.90, *p*=.10, partial-*η*^2^=.07). This effect suggests that in the AU task TRP tended to be generally higher during the HIP as compared to the LIP condition. Additionally, we observed significant effects of HEMI (*F*(1,39)=9.04, *p*=.01, partial-*η*^2^=.19) and AREA⁎HEMI (*F*(6,34)=5.49, *p*=.001, partial-*η*^2^=.49) showing that alpha power in the AU task was significantly higher in posterior parts of the right hemisphere than in the left hemisphere (*p*s<.001 in CT, CP, PT, PO regions) during both LIP and HIP condition (see Fig. 3).

### 3.3 Control analyses

We examined whether any of the observed TRP effects might be due to alpha power differences already present in the reference period or rather due to alpha power differences in the activation period (i.e., task period). To this end, we reran all ANOVAs using the logarithmized power of the reference and the activation periods that were used for calculation of the TRP as dependent variables. Concerning the significant TRP interaction effect COND⁎AREA⁎HEMI observed for the FS task, this effect was not evident in the reference period (*F*(6,34)=0.32, *p*=.92), but it was also found in the activation period (*F*(6,34)=2.77, *p*=.03). Concerning the significant TRP interaction effect AREA⁎HEMI observed in the AU task, we also found a significant interaction effect in the reference (*F*(6,34)=21.58, *p*<.001), but this effect was due to alpha lateralization (higher alpha power in the left vs. right hemisphere) in frontal brain regions (*p*s<.05 in AF, F, FC, CT regions) but not in posterior regions (*p*>.05 in CP, PT, PO regions) as it was found for TRP. Moreover, this interaction effect was also evident for the activation period (*F*(6,34)=33.60, *p*<.001) but this time including lateralization effects in posterior regions (*p*<.05, PO). Taken together, these analyses suggest that the observed TRP effects resulted from alpha power differences in the task itself rather than differences in the reference period.

We further examined whether the significant TRP level effect between tasks (main effect TASK) observed in the four-factorial ANOVA was due to alpha power differences in reference or activation periods. We found a significant main effect TASK in the reference (*F*(1,39)=6.34, *p*=.02) as well as in the activation period (*F*(1,39)=24.69, *p*<.001), indicating slightly higher reference alpha power in the FS as compared with the AU task (FS: 1.23; AU: 1.16; *Δ*=0.07), and lower activation alpha power in the FS as compared with the AU task (FS: 1.05; AU: 1.24; *Δ*=−0.19). This result pattern suggests that the TRP task effect is in part affected by differences in the reference period but more strongly driven by task differences in activation period.

For those interested in the effects of this experimental paradigm on the TRP in other EEG frequency bands we provide additional analyses for TRP in the theta and beta band in the Supplementary material. These analyses may be useful to determine to what extent the reported alpha TRP findings are specific for the alpha band.

## 4 Discussion

Behavioral analyses replicated the finding that enforcement of internal attention (HIP condition) impedes the performance in the four-word sentences (FS) task (cf., Benedek et al., 2011). This supports the assumption that the FS task typically involves bottom-up processing (sensory-intake), and becomes more difficult when it has to be performed without access to relevant external information. In contrast, as expected, the experimental manipulation did not affect task performance in the alternate uses (AU) task, supporting the assumption that the AU task intrinsically relies on internal attention (sensory-independence).

Looking at the EEG results, a significant condition effect on alpha TRP was observed for the FS task but not for the AU task. In other words, an enforcement of internal attention increased alpha power only in the sensory-intake task but not in the sensory-independence task. Moreover, the sensory-independence task generally showed higher task-related alpha power levels than the sensory-intake task in both experimental conditions. Control analyses showed that this task effect was mainly driven by alpha power differences during the task (i.e., activation period) and only to a minor degree of differences the reference period. This result pattern provides further empirical support for the hypothesis that alpha power increases as a function of internal attention demands. It is in line with previous studies comparing tasks with external vs. internal attention focus which consistently revealed higher alpha power for task with internal rather than external attention focus (Benedek et al., 2011; Cooper, Croft, Dominey, Burgess, & Gruzelier, 2003; Ray & Cole, 1985). Moreover, it is compatible with findings of alpha power increases during memory retention tasks where attention can be assumed to be directed internally (Jensen et al., 2002; Sauseng et al., 2005).

These findings may help to explain why divergent thinking has been consistently related to alpha synchronization (Fink & Benedek, 2013, in press), but was not observed in a study using the FS task when stimuli were unmasked (Benedek et al., 2011). Most divergent thinking tasks (e.g., alternate uses task, insight task, utopian situations task) involve brief verbal stimuli conveying conceptual information that can easily be retained in mind once it is encoded. In these tasks participants have to retrieve and recombine relevant semantic or episodic information to produce a creative response (Benedek, Franz, Heene, & Neubauer, 2012; Koestler, 1964). The four-word sentences task, however, can be seen as an exception as it requires considering four unrelated non-conceptual elements and hence benefits from continuous access to the external stimulus. Therefore, this task involves higher external attention demands than other divergent thinking tasks which results in lower task-related alpha power when external information can be accessed. Based on this rationale one can probably infer that alpha power increases can be especially observed in (divergent thinking) tasks that do not involve bottom-up processing even when the stimulus is visible. This is the case e.g., when the task could be performed equally well with eyes closed. This finding is particularly relevant for research suggesting that creative people show lower gating of external information or lower latent inhibition (Fink, Slamar-Halbedl, Unterrainer, & Weiss, 2012b; de Manzano, Cervenk, Karabanov, Farde, & Ullén, 2010).

Supplementary analyses explored whether similar effects can be observed in the theta or beta frequency band. In both frequency bands general effects of stimulus masking (higher task-related band power in HIP vs. LIP) were observed which were more pronounced in the AU than in the FS task. This result pattern thus is different from that observed in the alpha band, for which significant masking effects were observed particularly in the FS (sensory-intake) task, corresponding to the results of task performance. This suggests that the theta and beta band are sensitive to general processes associated with stimulus masking rather than to the distinction between internal/external information processes. These additional analyses hence can be seen as preliminary evidence for the specificity of the alpha band as indicator for the direction of attentional focus.

This study replicated the masking condition effect for the FS task (Benedek et al., 2011). As in the previous study, the condition effect was strongest over posterior parietal and occipital regions of the right hemisphere. It should be noted that in the previous study the condition effect was topographically less restricted and also applied to other regions of the brain. A possible reason for this difference is that the experimental condition (i.e., masked/unmasked stimuli) varied in the previous study between blocks of trials whereas it was fully randomized in this study. It is possible that the blocked presentation design of the previous study may have had a systematic effect on the reference period (e.g., expectation of masked trials in the HIP block could have involved more focused attention in order not to miss the stimulus which could have led to lower alpha power in the reference period). This could potentially have resulted in an overestimation of the synchronization effect in that earlier study; however, this study controlled for any potential expectation effects which may have resulted in topographically more specific condition effects, pointing to an important role of the right posterior brain regions for internal attention.

In all three conditions involving task-focused internal attention (FS task during HIP, and AU task during LIP and HIP), alpha activity showed a clearly lateralized pattern, with higher alpha power in posterior parietal and occipital regions of the right hemisphere. This lateralized TRP pattern is quite a consistent finding for divergent thinking (Fink Grabner, et al., 2009a; Fink Graif, et al., 2009; Fink & Benedek, in press; Jauk et al., 2012), and it has also been observed in other creative cognition tasks (Jung-Beeman et al., 2004; Schaefer, Vlek, & Desain, 2011). Moreover, stronger alpha power in right posterior regions has also been observed in studies outside the creativity domain (e.g., Ray and Cole, 1985; Jensen et al., 2002). Increased alpha over occipital–parietal sites has previously been interpreted as suppression of distracting information flow from the visual system (e.g., Jensen et al., 2002). While this interpretation is in line with the idea of focused internal attention, it does not explain why parietal alpha is more pronounced in the right hemisphere. Interestingly, fMRI studies employing divergent thinking tasks also commonly reported relatively lower activation (or deactivation) in right parietal regions (such as the right angular gyrus, and the right temporoparietal junction) during creative idea generation as compared to control tasks (Benedek et al., 2014a,b; Fink et al., 2009a, 2010, 2012a). Similar findings were also observed by fMRI studies on creative story generation (Howard-Jones, Blakemore, Samuel, Summers, & Claxton, 2005), designing of pens (Kowatari et al., 2009), or melodic improvisation in musicians (Berkowitz & Ansari, 2008, 2010). The right temporoparietal junction (rTPJ) and the ventral frontal cortex are part of a ventral attention network which is involved in the detection of behaviorally relevant sensory events (Corbetta, Patel, & Shulman, 2008; Corbetta & Shulman, 2002). Suppressed or attenuated activity in this region is thought to occur in response to top-down signals during goal-directed behavior in order to prevent reorienting of attention to task-irrelevant stimuli which would interfere with task performance. Specifically, frontal regions may exert top-down control over posterior regions by means of functional coupling between these brain regions (Klimesch, Sauseng, & Hanslmayr, 2007; Sauseng et al., 2005). These top-down signals serve a filtering function that shields goal-directed attention from distracting events. Although one generally has to be cautious when directly associating BOLD deactivation in fMRI with EEG alpha power (e.g., Gonçalves et al., 2006; Jann et al., 2009), the topographic coincidence between EEG and fMRI findings suggests that the alpha power increases in right-parietal regions during divergent thinking may correspond to deactivation of the rTPJ in fMRI studies, with both indicating the inhibition of the ventral attention network (cf., Fink & Benedek, 2013).

In divergent thinking, this mechanism may help to stay fully focused on internal processes such as retrieval from semantic and episodic memory during the performance of mental simulations and the construction of mental images (as during future thought; e.g., Schacter et al., 2012). The construction of novel mental images may underlie the more general process of imagination that is highly relevant for creative cognition. These internal processes are typically associated with activation of the default mode network (DMN). Recent fMRI studies thinking have also revealed consistent evidence for the relevance of DMN regions in divergent thinking (Benedek et al., 2014; Fink et al., 2012a). Further evidence for an association with trait creativity and the DMN comes from structural MRI studies and lesion studies (e.g., Fink et al., in press-a, in press-b; Jung et al., 2010; Shamay-Tsoory, Adler, Aharon-Peretz, Perry, and Mayseless, 2011). These studies suggest that creative cognition could be fruitfully understood in terms of the interplay of attention or control networks with intrinsic stimulus-independent networks such as the DMN (Jung, Mead, Carrasco, & Flores, 2013).

Inhibition of the ventral attentional network avoids attentional shifts to task-irrelevant stimuli, thus leading attention to stay focused during top-down, goal-driven tasks. It hence serves the function of task shielding during tasks requiring selective attention (e.g., Dreisbach and Haider, 2009). This mechanism may especially apply to tasks where attention is fully focused on internal processes, but it may not be exclusive to them and also include cognitive tasks requiring focused top-down processing of external information (e.g., Shulman et al., 2003). In this context it is important to consider that the inhibition of the ventral attention network does not take place in an all-or-nothing manner, but changes gradually in response to task demands. An fMRI study employing a memory retention paradigm showed that higher memory load was related to increased deactivation of the rTPJ (Todd, Fougnie, & Marois, 2005). Moreover, higher deactivation of the rTPJ was associated with better task performance in visual search (Shulman, Astafiev, McAvoy, d׳Avossa, & Corbetta, 2007). Similar findings were obtained in EEG studies showing that higher memory load was also related to stronger increases in alpha power (Klimesch et al., 1999; Jensen et al., 2002). This suggests that a more sensitive process (i.e., maintenance of a higher number of stimuli in short-term memory) requires a stronger shielding from distraction as evident in a stronger inhibition of the ventral attention network.

Considering all evidence, we propose that alpha power increases in right-parietal cortex reflect a gradual response corresponding to the strength of task-focused attention or task shielding, rather than merely indicating the direction of attention (internal vs. external). This notion is supported by the finding that the AU task showed significantly higher task-related alpha power than the FS task even in the HIP condition where no relevant external information was available and attention can only be focused on internal processes in both tasks. Moreover, alpha power increases cannot simply be attributed to higher task load, since the FS task involved a higher task load due to the letter-based processing of the stimulus. The AU task hence may represent a more sensitive process that requires a stronger focus of attention. Specifically, the AU task is known to involve different strategies such as the retrieval of old uses from memory (probably involving episodic memory), or imagining disassembling of the object for using or recombining parts of it (Gilhooly, Fioratou, Anthony, & Wynn, 2007). These imaginative processes include the generation and manipulation of mental images of possible uses. The generation of ideas in form of mental images (i.e., visual mental imagery; Kosslyn, Ganis, and Thompson (2001)) can be conceived as a very sensitive cognitive process that may be easily interfered by irrelevant sensory stimulation coming from the visual stream, and thus to benefit from task-focused attention (De Dreu, Nijstad, Bass, Wolsink, & Roskes, 2012). In contrast, the generation of original sentences in the FS task probably did not rely on figurative representations, but rather on the retrieval of relevant semantic information.

Along these lines, alpha power increases in right parietal cortex could also be considered as an indicator of the depth or elaborateness of an ongoing process of mental imagination (cf., Von Stein & Sarnthein, 2000), and thus represent a valid indicator of a cognitive process specific for creative cognition. This may not only explain alpha effects between tasks involving higher and lower amounts of divergent thinking (Fink, Benedek, Grabner, Staudt, & Neubauer, 2007), but also apply to individual differences in the ability to become immersed in a process of imagination. Effective executive processes are thought to be highly relevant for creative thought (Beaty & Silvia, 2012; Benedek & Neubauer, 2013; Benedek et al., 2012; De Dreu et al., 2012; Gilhooly et al., 2007; Jauk, Benedek, Dunst, & Neubauer, 2013; Jauk, Benedek, & Neubauer, 2014; Nusbaum & Silvia, 2011) and this may particularly involve the ability to keep attention focused on demanding internal processes such as idea generation and imagination.

## References

[bib1] Beaty R.E., Silvia P.J. (2012). Why do ideas get more creative across time? An executive interpretation of the serial order effect in divergent thinking tasks. Psychology of Aesthetics, Creativity and the Arts.

[bib7] Benedek M., Neubauer A.C. (2013). Revisiting Mednick׳s model on creativity-related differences in associative hierarchies. Evidence for a common path to uncommon thought. Journal of Creative Behavior.

[bib3] Benedek M., Bergner S., Könen T., Fink A., Neubauer A.C. (2011). EEG alpha synchronization is related to top-down processing in convergent and divergent thinking. Neuropsychologia.

[bib5] Benedek M., Franz F., Heene M., Neubauer A.C. (2012). Differential effects of cognitive inhibition and intelligence on creativity. Personality and Individual Differences.

[bib6] Benedek M., Mühlmann C., Jauk E., Neubauer A.C. (2013). Assessment of divergent thinking by means of the subjective top-scoring method: Effects of the number of top-ideas and time-on-task on reliability and validity. Psychology of Aesthetics, Creativity, and the Arts.

[bib4] Benedek M, Jauk E, Fink A, Koschutnig K, Reishofer G, Ebner G, Neubauer A. (2014). To create or to recall? Neural mechanisms underlying the generation of creative new ideas. NeuroImage.

[bib2] Benedek, M., Beaty, R., Jauk, E., Fink, A., Silvia, P. J., Dunst, B., & Neubauer, A.C. (2014a). Creating metaphors: The neural basis of figurative language production. *NeuroImage*, 90, 99-106, 10.1016/j.neuroimage.2013.12.046 10.1016/j.neuroimage.2013.12.046 PMC3951481 24384149

[bib8] Berkowitz A.L., Ansari D. (2008). Generation of novel motor sequences: The neural correlates of music improvisation. NeuroImage.

[bib9] Berkowitz A.L., Ansari D. (2010). Expertise-related deactivation of the right temporoparietal junction during musical improvisation. NeuroImage.

[bib10] Chakravarthi R., VanRullen R. (2012). Conscious updating is a rhythmic process. Proceedings of the National Academy of Sciences.

[bib11] Cooper N.R., Croft R.J., Dominey S.J. J., Burgess A.P., Gruzelier J.H. (2003). Paradox lost? Exploring the role of alpha oscillations during externally vs. internally directed attention and the implications for idling and inhibition hypotheses. International Journal of Psychophysiology.

[bib12] Corbetta M., Shulman G.L. (2002). Control of goal-directed and stimulus-driven attention in the brain. Nature Reviews Neuroscience.

[bib13] Corbetta M., Patel. G., Shulman G.L. (2008). The reorienting system of the human brain: From environment to theory of mind. Neuron.

[bib15] De Dreu C.K. W., Nijstad B.A., Bass M., Wolsink I., Roskes M. (2012). Working memory benefits creative insight, musical improvisation, and original ideation through maintained task-focused attention. Personality and Social Psychology Bulletin.

[bib14] de Manzano Ö., Cervenk S., Karabanov A., Farde L., Ullén F. (2010). Thinking outside a less intact box: Thalamic dopamine D2 receptor densities are negatively related to psychometric creativity in healthy individuals. PLoS One.

[bib16] Dreisbach G., Haider H. (2009). How task representations guide attention: Further evidence for the shielding function of task sets. Journal of Experimental Psychology: Learning, Memory, and Cognition.

[bib17] Engel A.K., Fries P., Singer W. (2001). Dynamic predictions: Oscillations and synchrony in top-down processing. Nature Reviews Neuroscience.

[bib18] Fink A., Benedek M., Vartanian O., Bristol A.S., Kaufman J.C. (2013). The creative brain: Brain correlates underlying the generation of original ideas. Neuroscience of creativity.

[bib19] Fink, A. & Benedek, M. (in press). EEG Alpha power and creative ideation. Neuroscience and Biobehavioral Reviews. Advance online publication. doi:10.1016/j.neubiorev.2012.12.002 10.1016/j.neubiorev.2012.12.002 PMC4020761 23246442

[bib27] Fink A., Neubauer A.C. (2006). EEG alpha oscillations during the performance of verbal creativity tasks: Differential effects of sex and verbal intelligence. International Journal of Psychophysiology.

[bib28] Fink A., Neubauer A.C. (2008). Eysenck meets Martindale: The relationship between extraversion and originality from the neuroscientific perspective. Personality and Individual Differences.

[bib21] Fink A., Grabner R.H., Benedek M., Neubauer A.C. (2006). Divergent thinking training is related to frontal electroencephalogram alpha synchronization. European Journal of Neuroscience.

[bib20] Fink A., Benedek M., Grabner R.H., Staudt B., Neubauer A.C. (2007). Creativity meets neuroscience: Experimental tasks for the neuroscientific study of creative thinking. Methods.

[bib22] Fink A., Grabner R.H., Benedek M., Reishofer G., Hauswirth V., Fally M., Neuper C., Ebner F., Neubauer A.C. (2009). The creative brain: Investigation of brain activity during creative problem solving by means of EEG and fMRI. Human Brain Mapping.

[bib24] Fink A., Graif B., Neubauer A.C. (2009). Brain correlates underlying creative thinking: EEG alpha activity in professional vs. novice dancers. NeuroImage.

[bib23] Fink A., Grabner R.H., Gebauer D., Reishofer G., Koschutnig K., Ebner F. (2010). Enhancing creativity by means of cognitive stimulation: Evidence from an fMRI study. NeuroImage.

[bib29] Fink A., Schwab D., Papousek I. (2011). Sensitivity of EEG upper alpha activity to cognitive and affective creativity interventions. International Journal of Psychophysiology.

[bib25] Fink A., Koschutnig K., Benedek M., Reishofer G., Ischebeck A., Weiss E.M., Ebner F. (2012). Stimulating creativity via the exposure to other people׳s ideas. Human Brain Mapping.

[bib30] Fink A., Slamar-Halbedl M., Unterrainer H. -F., Weiss E. (2012). Creativity: Genius, madness or a combination of both?. Psychology of Aesthetics, Creativity and the Arts.

[bib26] Fink A, Koschutnig K, Hutterer L, Steiner E, Benedek M, Weber B, Reishofer G, Papousek I, Weiss EM (in press-a). Gray matter density in relation to different facets of verbal creativity. Brain Structure & Function. doi:10.1007/s00429-013-0564-0 10.1007/s00429-013-0564-0 23636224

[bib31] Fink, A., Weber, B., Koschutnig, K., Benedek, M., Reishofer, G., Ebner, F., Papousek, I., & E. M. Weiss (in press-b). Creativity and schizotypy from the neuroscience perspective. Cognitive, Affective, & Behavioral Neuroscience. doi:10.3758/s13415-013-0210-6 10.3758/s13415-013-0210-6 24022793

[bib32] Fries P. (2005). A mechanism for cognitive dynamics: Neuronal communication through neuronal coherence. Trends in Cognitive Sciences.

[bib33] Gilhooly K.J., Fioratou E., Anthony S.H., Wynn V. (2007). Divergent thinking: Strategies and executive involvement in generating novel uses for familiar objects. British Journal of Psychology.

[bib505] Gonçalves S.I., deMunck J.C., Pouwels P.J.W., Schoonhoven R., Kuijer J.P.A., Maurits N.M., Hoogduin J.M., VanSomeren E.J.W., Heethaar R.M., LopesdaSilva F.H. (2006). Correlating the alpha rhythm to BOLD using simultaneous EEG/fMRI: Inter-subject variability. NeuroImage.

[bib34] Grabner R.H., Fink A., Neubauer A.C. (2007). Brain correlates of self-rated originality of ideas: Evidence from event-related power and phase-locking changes in the EEG. Behavioral Neuroscience.

[bib35] Gratton G., Coles M.G., Donchin E. (1983). A new method for off-line removal of ocular artifact. Electroencephalography and Clinical Neurophysiology.

[bib36] Guilford J.P. (1967). The nature of human intelligence.

[bib37] Händel B.F., Haarmeier T., Jensen O. (2011). Alpha oscillations correlate with the successful inhibition of unattended stimuli. Journal of Cognitive Neuroscience.

[bib38] Howard-Jones P.A., Blakemore S.-J., Samuel E.A., Summers I.R., Claxton G. (2005). Semantic divergence and creative story generation: An fMRI investigation. Cognitive Brain Research.

[bib39] Jann K., Dierks T., Boesch C., Kottlow M., Strik W., Koenig T. (2009). BOLD correlates of EEG alpha phase-locking and the fMRI default mode network. NeuroImage.

[bib42] Jauk E., Benedek M., Neubauer A.C. (2012). Tackling creativity at its roots: Evidence for different patterns of EEG alpha activity related to convergent and divergent modes of task processing. International Journal of Psychophysiology.

[bib40] Jauk E., Benedek M., Dunst B., Neubauer A.C. (2013). The relationship between intelligence and creativity: New support for the threshold hypothesis by means of empirical breakpoint detection. Intelligence.

[bib41] Jauk, E., Benedek, M., & Neubauer, A.C. (2014). The road to creative achievement: A latent variable model of ability and personality predictors. European Journal of Personality, 28, 95-105, doi:10.1002/per.1941 10.1002/per.1941 PMC3923982 24532953

[bib43] Jaušovec N. (1997). Differences in EEG activity during the solution of closed and open problems. Creativity Research Journal.

[bib44] Jaušovec N. (2000). Differences in cognitive processes between gifted, intelligent, creative, and average individuals while solving complex problems: An EEG Study. Intelligence.

[bib47] Jensen O., Mazaheri A. (2010). Shaping functional architecture by oscillatory alpha activity: Gating by inhibition. Frontiers in Human Neuroscience.

[bib46] Jensen O., Gelfand J., Kounios J., Lisman J.E. (2002). Oscillations in the alpha band (9–12 Hz) increase with memory load during retention in a short-term memory task. Cerebral Cortex.

[bib45] Jensen O., Bonnefond M., VanRullen R. (2012). An oscillatory mechanism for prioritizing salient unattended stimuli. Trends in Cognitive Sciences.

[bib49] Jung R.E., Segall J.M., Bockholt H.J., Flores R.A., Smith S.M., Chavez R.S., Haier R.J. (2010). Neuroanatomy of creativity. Human Brain Mapping.

[bib48] Jung R.E., Mead B.S., Carrasco J., Flores R.A. (2013). The structure of creative cognition in the human brain. Frontiers in Human Neuroscience.

[bib50] Jung-Beeman M., Bowden E.M., Haberman J., Frymiare J.L., Arambel-Liu S., Greenblatt R., Reber P.J., Kounios J. (2004). Neural activity when people solve verbal problems with insight. PLoS Biology.

[bib51] Kaufman J.C., Plucker J.A., Baer J (2008). Essentials of creativity assessment.

[bib52] Klimesch W. (1999). EEG alpha and theta oscillations reflect cognitive and memory performance: A review and analysis. Brain Research Reviews.

[bib53] Klimesch W. (2012). Alpha-band oscillations, attention, and controlled access to stored information. Trends in Cognitive Sciences.

[bib54] Klimesch W., Doppelmayr M., Schwaiger J., Auinger P., Winkler T. (1999). “Paradoxical” alpha synchronization in a memory task. Cognitive Brain Research.

[bib55] Klimesch W., Sauseng P., Hanslmayr S. (2007). EEG alpha oscillations: The inhibition-timing hypothesis. Brain Research Reviews.

[bib56] Koestler A. (1964). The act of creation.

[bib57] Kosslyn S.M., Ganis G., Thompson W.L. (2001). Neural foundations of imagery. Nature Reviews Neuroscience.

[bib58] Kowatari Y., Lee S.H., Yamamura H., Nagamori Y., Levy P., Yamane S., Yamamoto M. (2009). Neural networks involved in artistic creativity. Human Brain Mapping.

[bib59] Martindale C., Hasenfus N. (1978). EEG differences as a function of creativity, stage of the creative process, and effort to be original. Biological Psychology.

[bib60] Martindale C., Hines D. (1975). Creativity and cortical activation during creative, intellectual, and EEG feedback tasks. Biological Psychology.

[bib61] Niedermeyer E., Lopes da Silva F.H. (1999). Electroencephalography: Basic Principles, Cinical Applications, and Related Fields.

[bib62] Nusbaum E.C., Silvia P.J. (2011). Are intelligence and creativity really so different? Fluid intelligence, executive processes, and strategy use in divergent thinking. Intelligence.

[bib63] Pfurtscheller G., Pfurtscheller G., Lopes da Silva F.H. (1999). Quantification of ERD and ERS in the time domain.

[bib64] Pfurtscheller G., Stancak A., Neuper C. (1996). Event-related synchronization (ERS) in the alpha band—an electrophysiological correlate of cortical idling: A review. International Journal of Psychophysiology.

[bib65] Ray W.J., Cole H.W. (1985). EEG alpha reflects attentional demands, and beta activity reflects emotional and cognitive processes. Science.

[bib66] Rihs T.A., Michel C.M., Thut G. (2007). Mechanisms of selective inhibition in visual spatial attention are indexed by α-band EEG synchronization. European Journal of Neuroscience.

[bib67] Sauseng P., Klimesch W., Doppelmayr M., Pecherstorfer T., Freunberger R., Hanslmayr S. (2005). EEG alpha synchronization and functional coupling during top-down processing in a working memory task. Human Brain Mapping.

[bib68] Schacter D.L., Addis D.R., Hassabis D., Martin V.C., Spreng R.N., Szpunar K.K. (2012). The future of memory: Remembering, imagining, and the brain. Neuron.

[bib69] Schaefer R.S., Vlek R.J., Desain P. (2011). Music perception and imagery in EEG: Alpha band effects of task and stimulus. International Journal of Psychophysiology.

[bib70] Schoppe K. (1975). Verbaler Kreativitäts-Test [Verbal creativity test] (V-K-T).

[bib71] Shamay-Tsoory S.G., Adler N., Aharon-Peretz J., Perry D., Mayseless N. (2011). The origins of originality: The neural bases of creative thinking and originality. Neuropsychologia.

[bib72] Shulman G.L., McAvoy M.P., Cowan M.C., Astafiev S.V., Tansy A.P., d׳Avossa G., Corbetta M. (2003). Quantitative analysis of attention and detection signals during visual search. Journal of Neurophysiology.

[bib73] Shulman G.L., Astafiev S.V., McAvoy M.P., d׳Avossa G., Corbetta M. (2007). Right TPJ deactivation during visual search: Functional significance and support for a filter hypothesis. Cerebral Cortex.

[bib74] Todd J.J., Fougnie D., Marois R. (2005). Visual short-term memory load suppresses temporo-parietal junction activity and induces inattentional blindness. Psychological Science.

[bib75] Vasey M.W., Thayer J.F. (1987). The continuing problem of false positives in repeated measures ANOVA in psychophysiology: A multivariate solution. Psychophysiology.

[bib76] Von Stein A., Sarnthein J. (2000). Different frequencies for different scales of cortical integration: From local gamma to long range alpha/theta synchronization. International Journal of Psychophysiology.

[bib77] Worden M., Foxe J.J., Wang N., Simpson G.V. (2000). Anticipatory biasing of visuospatial attention indexed by retinotopically specific α-Band electroencephalography increases over occipital cortex. Journal of Neuroscience.

[bib78] Zanto T.P., Rubens M.T., Thangavel A., Gazzaley A. (2011). Causal role of the prefrontal cortex in top-down modulation of visual processing and working memory. Nature Neuroscience.

