# Supplementary material for: Alpha power increases in right parietal cortex reflects focused internal attention
Source: Neuropsychologia. 2014 Apr;56(100):393–400. doi: 10.1016/j.neuropsychologia.2014.02.010 (PMC3989020; doi:10.1016/j.neuropsychologia.2014.02.010)
Supplement: Supplementary file 1 — Supplementary data [file mmc1.doc]

**Supplementary Materials**

**Complementary analyses for the theta frequency band (4.5-6.5 Hz)**

As for the alpha band, we first run a 2x2x2x7 ANOVA considering the within-subject factors TASK (FS, sensory-intake vs. AU, sensory-independence), experimental condition (COND: LIP vs. HIP), hemisphere (HEMI: left vs. right) and AREA (anteriofrontal, frontal, frontocentral, centrotemporal, centroparietal, parietotemporal, and parietooccipital). This analysis showed a significant TASK*AREA interaction (*F*[6,34] = 3.44, *p* = .01, partial-η^2^ = .38) indicating higher task-related theta synchronization especially in frontal sites during the FS as compared to the AU task. The analysis did, however, not reveal any significant TRP effects related to the experimental condition besides a tendency towards generally higher task-related theta synchronization in the HIP (vs. LIP) condition (COND: *F*[1,39] = 3.26, *p* = .08, partial-η^2^ = .08).

We further explored effects for each task separately. For the FS task we observed no significant TRP effects related to the experimental condition. For the AU task, however, we observed a significant main effect of condition (COND: *F*[1,39] = 4.53, *p* = .04, partial-η^2^ = .10) indicating a stronger task-related theta synchronization in the HIP condition. This result pattern thus is unlike that obtained for the alpha frequency range where a significant interaction of COND*AREA*HEMI was observed for the FS task, but no significant effect related to COND in the AU task.

**Complementary analyses for the beta frequency band (15.5-30.5 Hz)**

The same analyses were run for the TRP in the beta band. The four-factorial ANOVA revealed a significant effect of TASK (*F*[1,39] = 25.15, *p* < .001, partial-η^2^ = .39) which was further moderated by AREA (TASK*AREA: *F*[6,34] = 6.11, *p* < .001, partial-η^2^ = .52) indicating stronger task-related desynchronization in the beta band during the FS as compared to the AU task especially at central and posterior brain regions. Moreover, we observed a significant interaction of COND*AREA (*F*[6,34] = 2.58, *p* = .04, partial-η^2^ = .31) suggesting lower task-related beta desynchronization during the HIP as compared to the LIP condition restricted to posterior regions of the brain.

Analyses focusing on the FS task again revealed lower task-related beta desynchronization during the HIP (vs. LIP) condition at posterior regions of the brain (COND*AREA: *F*[6,34] = 2.96, *p* = .02, partial-η^2^ = .34; Tukey’s HSD posttests showed a marginally significant difference at parietotemporal regions, *p* = .08). In the AU task, a lateralization towards lower beta desynchronization in the right hemisphere was observed in the HIP condition (*p* < .001) but not in the LIP condition (*p* = .93; COND*HEMI: *F*[1,39] = 7.56, *p* = .01, partial-η^2^ = .16).
